# Domain-Specific Associations Between Physical Activity and Tinnitus in NHANES 2015–2018

**DOI:** 10.3390/audiolres16030090

**Published:** 2026-06-12

**Authors:** Mitra Britton, Peter A. Hosick

**Affiliations:** 1 Department of Communication Sciences and Disorders, Montclair State University, Montclair, NJ 07043, USA; 2 Department of Kinesiology, Montclair State University, Montclair, NJ 07043, USA; hosickp@montclair.edu

**Keywords:** tinnitus, NW PA = non-work-related physical activity, WORK PA = work-related physical activity, NHANES

## Abstract

Background/Objectives: Tinnitus is a prevalent auditory condition associated with significant psychological burden and limited treatment efficacy. While physical activity confers broad health benefits, its relationship with tinnitus remains understudied. This study examined associations between domain-specific physical activity and tinnitus in a nationally representative sample of U.S. adults. Methods: Data from NHANES 2015–2018 were analyzed. The final analytic sample comprised 4301 adults aged 20 years and older. Tinnitus was assessed via the NHANES audiometry questionnaire. Physical activity was categorized as low, moderate, and high (MET-min/week) separately for work-related (WORK) and non-work-related (NW) domains. Survey-weighted multivariable logistic regression models, adjusted for age, sex, race, BMI, poverty–income ratio, sedentary time, smoking, education, noise exposure, hypertension, diabetes, and depressive symptoms, were used to examine associations. Linear trends across ordered physical activity categories were evaluated using ordinal trend analyses. Results: The weighted prevalence of tinnitus was 17.3%. High NW physical activity (PA) was associated with significantly lower odds of tinnitus (OR = 0.70, 95% CI: 0.488–0.995, *p* = 0.0475), while high WORK PA was associated with significantly higher odds (OR = 1.30, 95% CI: 1.06–1.60, *p* = 0.018). Trend analyses confirmed opposing linear trends across ordered categories: inverse for NW PA (OR = 0.83 per category, *p*-trend = 0.0406) and positive for WORK PA (OR = 1.14 per category, *p*-trend = 0.017). Noise exposure and depressive symptoms were independently associated with tinnitus across both models. Conclusions: These findings suggest a domain-specific paradox: NW PA was associated with lower odds of tinnitus, whereas WORK PA was associated with higher odds. These results highlight the importance of domain-specific assessment and identify recreational activity as a potential modifiable factor warranting further investigation. Given the cross-sectional design, these associations should not be interpreted as causal.

## 1. Introduction

Tinnitus, the perception of sound in the absence of an external acoustic stimulus, is one of the most prevalent chronic auditory conditions in the United States, affecting an estimated 10% of adults [1]. Beyond its auditory dimensions, tinnitus is associated with significant psychological comorbidities, including anxiety, depression, and sleep disturbance, all of which contribute to reduced quality of life [2]. Despite its prevalence and multidimensional burden, current treatment options offer limited efficacy, and the modifiable behavioral factors that influence tinnitus onset and severity remain poorly understood [2].

There is a substantial body of scientific evidence supporting the benefits of physical activity, including reductions in all-cause mortality and improvements in cardiovascular health, cognitive function, and well-being [3,4]. This raises the question of whether physical activity could modulate tinnitus perception. Understanding this relationship is important, as physical activity is a non-invasive behavior that may be relevant to tinnitus management. However, limited research has examined the relationship between physical activity and tinnitus.

Although research in this area remains sparse, the available evidence points toward a possible inverse link between physical activity and tinnitus [5,6,7,8]. Using the National Health and Nutrition Examination Survey (NHANES) data, Loprinzi et al. (2013) reported that each additional minute of daily moderate-to-vigorous activity corresponded to a 4% reduction in the likelihood of tinnitus lasting longer than three months among adolescents, consistent with an inverse association [6]. Chen et al. (2023) similarly observed that tinnitus was less frequently reported among physically active individuals than inactive ones (12.8% versus 18.5%); in adjusted subgroup analyses, weekly activity volumes of 150–300, 310–540, and 550–4800 min were each associated with reduced odds of tinnitus (OR = 0.72, 0.56, and 0.62, respectively) [7]. In a cross-sectional sample of 3004 adults (2751 with tinnitus and 253 controls), Chalimourdas et al. (2025) found that accumulating more than 150 min of weekly leisure-time moderate-to-vigorous activity was linked to lower odds of tinnitus (OR = 0.515, *p* < 0.001), whereas sitting for more than seven hours daily was linked to higher odds (OR = 2.366, *p* < 0.001) [5]. Beyond prevalence, Carpenter-Thompson et al. reported that greater physical activity related to less severe tinnitus and improved quality of life among affected adults; although the effects were modest, activity and tinnitus severity each contributed independently to quality of life [8].

Previous studies have largely focused on recreational physical activity, with limited attention to other important domains, such as occupational activity. This narrow focus may obscure domain-specific associations and lead to an incomplete understanding of how physical activity relates to tinnitus. Different physical activity domains may have distinct physiological, environmental, and psychosocial correlates, suggesting that their relationships with tinnitus may vary [9]. Therefore, evaluating physical activity across multiple domains may provide a more nuanced assessment of its relationship with tinnitus.

Given the high prevalence of tinnitus and the limited effectiveness of current treatment options, identifying modifiable factors associated with tinnitus is of substantial public health interest. This study utilized data from NHANES, a large, nationally representative sample of U.S. adults, to examine the associations between domain-specific physical activity, including both work-related and non-work-related activities, and tinnitus. This study further assessed dose–response relationships by testing for linear trends across ordered physical activity categories within each domain, thereby providing insight into the potential relevance of physical activity to tinnitus.

## 2. Materials and Methods

### 2.1. Participants

**Figure 1:**
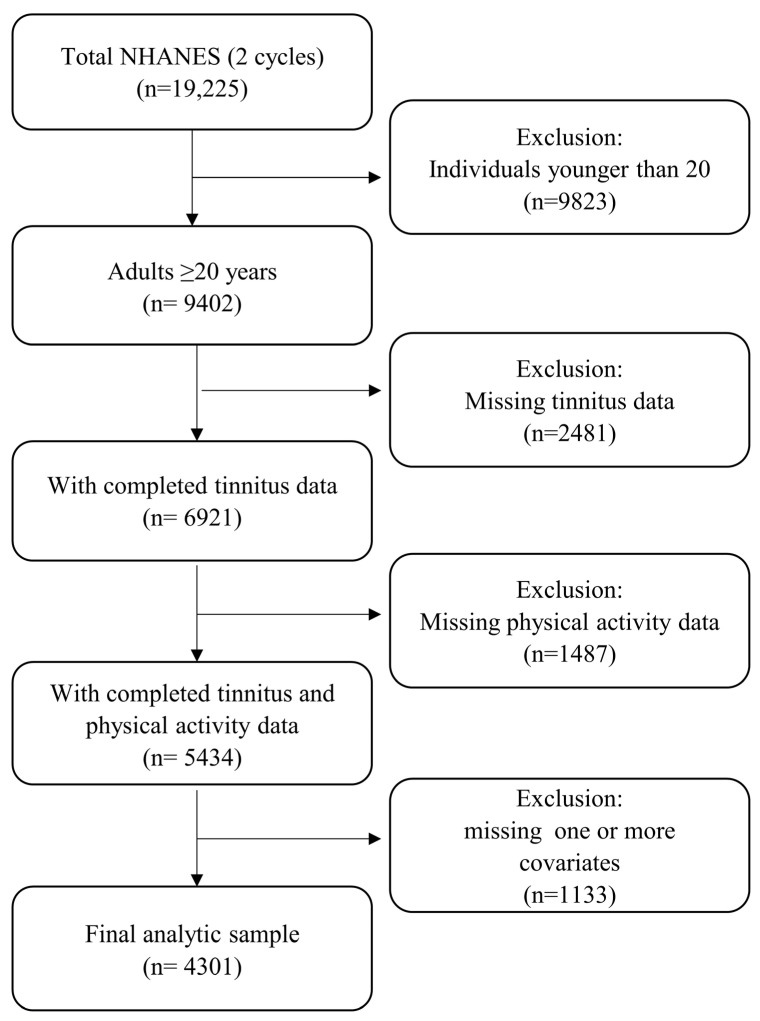
Flowchart of participant selection and exclusion criteria for the analytic sample.

Data for this study were drawn from NHANES, a program administered every two years by the Centers for Disease Control and Prevention (CDC). NHANES characterizes the noninstitutionalized civilian population of the United States using a complex, multistage probability sampling approach. The 2015–2016 and 2017–2018 NHANES datasets were analyzed. All NHANES protocols receive approval from the CDC research ethics review board, and participants provide written informed consent prior to data collection. The initial combined sample comprised 19,225 participants across both cycles. Participants younger than 20 years were excluded (n = 9823), leaving 9402 eligible participants. Of these, 2481 were further excluded for missing or incomplete responses to the tinnitus question, leaving 6921 participants with a valid tinnitus status. Subsequently, 1487 participants were excluded due to missing data on one or more physical activity variables, including work-related physical activity, recreational physical activity, and transportation physical activity, yielding 5434 participants. Finally, 1133 participants were excluded due to missing data on one or more covariates. The final analytic sample comprised 4301 participants. The study inclusion and exclusion process is illustrated in Figure 1.

### 2.2. Physical Activity Assessment

Physical activity was measured using the NHANES Physical Activity Questionnaire, which is based on the Global Physical Activity Questionnaire (GPAQ). For each of three domains, including occupational (work-related), recreational, and transportation-related activities (walking or bicycling for transportation), participants indicated how many days per week and how many minutes per day they engaged in moderate- and vigorous-intensity activity. Following NHANES analytic guidelines, weekly energy expenditure was expressed in metabolic equivalent (MET) minutes, assigning 8 METs to vigorous activity and 4 METs to moderate-intensity and transportation activity. Domain-specific physical activity variables were computed by multiplying MET values by the reported days per week and minutes per day, then summing across relevant components. Work-related physical activity (WORK PA) was determined as the sum of vigorous and moderate occupational activity. Non-work-related physical activity (NW PA) was determined as the sum of vigorous recreational, moderate recreational, and transportation-related activity. Domain-specific physical activity variables were classified into low (<600 MET-min/week), moderate (600–1200 MET-min/week), and high (>1200 MET-min/week) levels based on established physical activity guidelines [10]. These cut points correspond to the lower and upper bounds of the WHO-recommended weekly activity volume [10]. Because MET-min/week is computed identically across domains, these volume-based thresholds were applied within each domain to enable a consistent comparison of activity volume, and were not intended to equate the physiological effects of occupational and leisure activity, which differ in intensity, duration, and postural demands [11]. Sedentary behavior was evaluated through self-reported daily sitting time (minutes per day) and was included as a covariate in multivariable models. For analysis and reporting, sedentary time was converted from minutes to hours per day to improve interpretability.

### 2.3. Tinnitus Assessment

The NHANES audiometry questionnaire was used to assess participants’ history of tinnitus. Participants were asked whether, in the past 12 months, they had been bothered by tinnitus, ringing, or buzzing in the ears lasting five minutes or more, with response options of ‘yes’ or ‘no’. Individuals who answered ‘yes’ were classified as having a history of tinnitus.

### 2.4. Covariates

Sociodemographic, behavioral, environmental, and health-related variables associated with both physical activity and tinnitus were included as covariates to minimize potential confounding. All covariates were selected a priori based on existing literature. These variables include age, sex, race, education level, poverty–income ratio (PIR), body mass index (BMI), smoking status, noise exposure, depressive symptoms, hypertension, and diabetes. Race was categorized as Mexican American, other Hispanic, non-Hispanic White, non-Hispanic Black, and other race (including multiracial). Educational attainment was classified into high school or below, some college or an associate degree, and a college graduate or above. Body mass index was derived as weight in kilograms over the square of height in meters. The income-to-poverty ratio was modeled continuously, with higher values denoting greater socioeconomic advantage [12], and participants were grouped by smoking history as never, former, or current smokers [13]. Noise exposure was defined as a positive response to either occupational or non-occupational noise exposure. Occupational noise exposure was assessed by the question, “Have you ever had a job or combination of jobs where you were exposed to loud sounds or noise for 4 or more hours per day, several days per week?” Non-occupational noise exposure was assessed by the question, “Outside of a job, have you ever been exposed to very loud noise or music for 10 or more hours per week?” [7]. Depressive symptoms were assessed with the Patient Health Questionnaire-9, on which each of the nine items is rated from 0 (“not at all”) to 3 (“nearly every day”). Summed scores span 0 to 27, and a threshold of ≥10 was applied to indicate clinically significant symptoms [14]. Hypertension status was determined based on self-reported physician diagnosis of hypertension or high blood pressure. Diabetes status was defined by a self-reported diagnosis of diabetes, excluding gestational diabetes.

### 2.5. Statistical Analysis

All analyses were conducted in accordance with NHANES analytic guidelines. To generate nationally representative estimates, all analyses incorporated the strata, primary sampling units, and examination weights that reflect the survey’s complex multistage design. When multiple NHANES cycles were combined, examination weights were appropriately rescaled according to NHANES recommendations. Descriptive characteristics of participants were summarized using unweighted counts with survey-weighted percentages for categorical variables and survey-weighted means with standard errors for continuous variables, stratified by tinnitus status. Group differences were assessed using design-based Rao–Scott chi-square tests for categorical variables and design-based *t*-tests for continuous variables. All descriptive analyses incorporated the NHANES weights and complex sampling design.

Survey-weighted multivariable logistic regression models were used to examine the associations between physical activity and tinnitus. Physical activity was assessed separately by domain, and two primary models were fitted: one including WORK PA and one including NW PA (transportation and recreational). Physical activity was categorized into low, moderate, and high levels based on weekly metabolic equivalent (MET) minutes. Models were adjusted for age, sex, race, PIR, BMI, sedentary time, smoking status, education level, noise exposure, hypertension, diabetes, and depressive symptoms. Additionally, tests for linear trend across increasing levels of physical activity were performed by modeling physical activity categories as ordinal variables. Odds ratios (ORs) and 95% confidence intervals (CIs) were reported. All statistical tests were two-sided, and *p*-values < 0.05 were considered statistically significant. Statistical analyses were performed using R (version 4.5.0).

## 3. Results

### 3.1. Study Sample

A total of 4301 adults aged 20 years and older were included in the final analytic sample after applying complete-case restrictions across all study variables. The weighted prevalence of tinnitus was 17.3% (SE = 0.01). The weighted mean age of the sample was 47.9 years (SE = 0.48). Regarding WORK PA, 58.0% of participants were classified as Low, 7.2% as Moderate, and 34.8% as High. For NW PA, 53.4% were classified as Low, 16.8% as Moderate, and 29.8% as High. Detailed characteristics of the study sample are presented in Table 1.

### 3.2. Association Between WORK PA and Tinnitus

In the multivariable-adjusted model, participants in the Moderate WORK PA category did not differ significantly in tinnitus odds compared to those in the Low category (OR = 0.83, 95% CI: 0.47–1.46, *p* = 0.476). However, participants in the high WORK PA category had significantly higher odds of tinnitus than those in the Low category (OR = 1.30, 95% CI: 1.06–1.60, *p* = 0.018).

Several covariates were significantly associated with tinnitus in this model. Noise exposure was independently associated with approximately twice the odds of tinnitus (OR = 1.95, *p* = 0.002). Older age was positively associated with tinnitus (OR = 1.02 per year, *p* < 0.001). Depression was associated with significantly higher odds of tinnitus (OR = 2.03, *p* = 0.001). Greater sedentary time was associated with modestly increased odds of tinnitus (OR = 1.04 per hour, 95% CI: 1.002–1.082, *p* = 0.038), though the effect size was small. Compared to Mexican American participants, Non-Hispanic Black participants (OR = 0.52, *p* = 0.001) and Other Hispanic participants (OR = 0.61, *p* = 0.015) had significantly lower odds of tinnitus. Poverty–income ratio, Sex, BMI, education, smoking status, hypertension, and diabetes were not significantly associated with tinnitus in this model. Table 2 presents the detailed information.

### 3.3. Association Between NW PA and Tinnitus

For NW PA, no significant association was observed between the Moderate category and tinnitus relative to the Low category (OR = 0.79, 95% CI: 0.53–1.17, *p* = 0.211). Participants classified in the high NW PA category had significantly lower odds of tinnitus than those in the Low category (OR = 0.70, 95% CI: 0.488–0.995, *p* = 0.0475).

In this model, a similar pattern of covariate associations was observed. Noise exposure remained significantly associated with tinnitus (OR = 2.02, *p* = 0.001). Older age was positively associated with tinnitus (OR = 1.021 per year, *p* < 0.001). Depression was associated with significantly higher odds of tinnitus (OR = 1.98, *p* = 0.002). Compared to Mexican American participants, Non-Hispanic Black participants (OR = 0.54, *p* = 0.001) and Other Hispanic participants (OR = 0.62, *p* = 0.019) had significantly lower odds of tinnitus. Unlike the WORK PA model, sedentary time was not significantly associated with tinnitus in the NW PA model (*p* = 0.148). Sex, BMI, PIR, education, smoking status, hypertension, and diabetes were not significantly associated with tinnitus in this model. Table 3 presents the detailed information.

### 3.4. Trend Analysis

To further evaluate the nature of the association between physical activity and tinnitus, tests for linear trend across ordered categories were conducted by modeling physical activity as an ordinal variable across the three categories (Low = 1, Moderate = 2, High = 3) within each domain. The results revealed opposing and statistically significant linear trends for WORK PA and NW PA.

**Figure 2:**
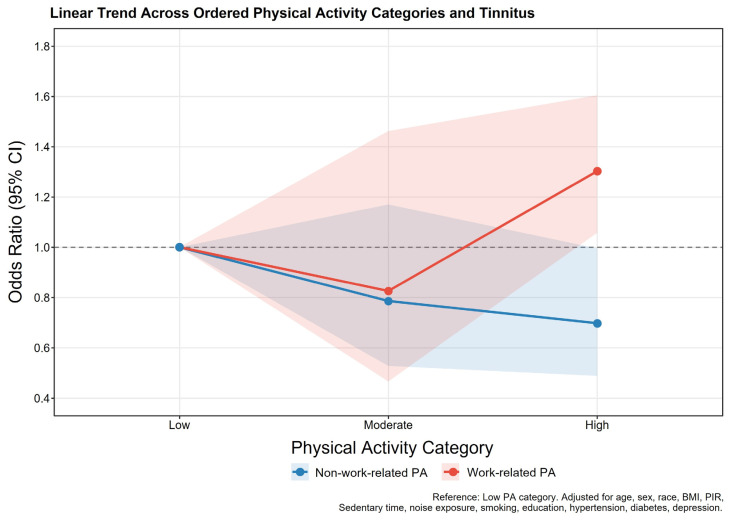
Linear trend across ordered physical activity categories and tinnitus prevalence among US adults (NHANES 2015–2018). Adjusted odds ratios (95% CI) for tinnitus are shown across Low, Moderate, and High physical activity categories for WORK PA work-related (red) and NW PA (blue) physical activity. The Low category serves as the reference (OR = 1.00). Shaded bands represent 95% CIs. The dashed line indicates no association. Models were adjusted for age, sex, race, BMI, poverty–income ratio, sedentary time, noise exposure, smoking, education, hypertension, diabetes, and depression. PA = physical activity; OR = odds ratio; CI = confidence interval.

For NW PA, a statistically significant inverse linear trend was identified (β = −0.186, OR per category increase = 0.83, 95% CI: 0.70–0.99, *p*-trend = 0.0406), indicating that each successive step increase in NW PA category was independently associated with 17% lower odds of tinnitus. In contrast, a statistically significant positive linear trend was observed for WORK PA (β = 0.126, OR per category increase = 1.14, 95% CI: 1.03–1.25, *p*-trend = 0.017). The trend test corresponded to an average per-category increase of approximately 14% in the odds of tinnitus. The detailed results of the trend analyses are presented in Table 4, and the linear trends across ordered categories for both physical activity domains are illustrated in Figure 2.

## 4. Discussion

This study aimed to examine the associations between tinnitus and domain-specific physical activity in U.S. adults. The primary findings indicate a paradoxical pattern: there is an inverse relationship between NW PA and tinnitus, although this association was borderline and should be interpreted with caution, whereas increased WORK PA is associated with higher tinnitus prevalence. Although limited, previous work exploring physical activity and tinnitus has not explored domain-specific effects. Thus, this study represents the first report of a paradox between physical activity type and tinnitus. This concept, however, is supported by several recent studies that also found differential responses to WORK PA vs. leisure-time physical activity with respect to cardiovascular disease [9], inflammation [15], and chronic stress [16].

Like in many chronic conditions, physical activity has been associated with reduced severity and prevalence of tinnitus [5,6]. For example, a recent study reported that more intense leisure-time PA was associated with lower tinnitus loudness and severity. They extended their findings by demonstrating that sedentary behavior was also associated with higher odds of tinnitus [5]. The present investigation found a similar relationship, as greater high physical activity was associated with lower tinnitus prevalence. We found that high WORK PA, compared with low WORK PA, was associated with higher odds of tinnitus. It should be noted that the Moderate WORK PA category produced an imprecise, non-significant estimate (OR = 0.83, 95% CI: 0.47–1.46, *p* = 0.476), reflecting the small size of this subgroup (n = 249, 7.2% of the sample). The positive linear trend is therefore attributable primarily to the High vs. Low contrast rather than a consistent step-wise increase across categories. Unlike previous work, this analysis separated work and NW PA, uncovering a paradox between the two domains. This important distinction may explain why total physical activity measures can obscure meaning differences between physical activity domains.

The physiologic effect of WORK PA or occupational PA and non-work physical activity may not be equivalent [9,17]. While mechanisms explaining the physical activity paradox are not fully elucidated, potential mechanisms relevant here include the idea that occupational physical activity is too long or of low intensity to improve health and may even impair cardiovascular health [18,19]. This is relevant because impaired cardiovascular health is associated with increased tinnitus prevalence and severity [20,21,22]. Occupational physical activity often involves repeated lifting heavy and or static postures, which can increase blood pressure [23]. Hypertension prevalence has previously been shown to be approximately 12% higher in those with tinnitus than in those without [24]. In the present study, 45.4% of those with tinnitus had hypertension compared to 30.3% of those without tinnitus, although hypertension was not independently associated with tinnitus in the adjusted models. Lastly, occupational physical activity can increase baseline inflammation [15,25]. With regard to tinnitus, it is not entirely clear whether inflammation is the cause or effect of tinnitus; however, pharmacologic intervention to reduce inflammatory markers may be effective for tinnitus treatment [26], suggesting inflammation may be involved in the pathophysiology of tinnitus. While inflammatory markers were not assessed in the present investigation, this is an area for further research into the development and prevention of tinnitus. Collectively, this suggests cardiovascular, hypertensive, and inflammatory pathways may contribute to the association between occupational activity and tinnitus.

Additional secondary factors may also influence tinnitus prevalence in those with high physical activity at work. Although included as a covariate in the present investigation, occupational noise exposure has been recognized as a contributor to a host of hearing issues, including tinnitus [27]. Current data are consistent with this, as noise exposure was more common among participants with tinnitus than those without (55.4% vs. 38.0%). However, this measure is self-reported and is likely to under-capture true occupational noise exposure, which may explain why residual confounding by noise exposure could persist even after statistical adjustment. Other secondary factors, including air pollution [28] and diet [29], have shown mixed findings regarding their influence on tinnitus [28,29,30]. Nevertheless, exposure to air pollutants and dietary choices are often influenced by occupation, along similar lines that separate those with WORK PA from those without. Using larger population-based data may help to explore these relationships more closely.

An additional finding warranting comment was that, relative to Mexican American participants, Non-Hispanic Black and Other Hispanic participants had significantly lower adjusted odds of tinnitus in both models. This is broadly consistent with prior population-based work documenting racial and ethnic differences in tinnitus prevalence among U.S. adults, including a higher reported prevalence in Non-Hispanic White adults relative to several minority groups [1]. The mechanisms underlying these differences are not well understood and may reflect variation in noise exposure, audiometric thresholds, healthcare access, or differences in symptom reporting. As these patterns were not a primary focus of the present analysis, they should be interpreted with caution and represent an area for dedicated future investigation.

The current investigation is not without several limitations. First, the cross-sectional design limits the findings to associations rather than causality. However, the findings uncover a new paradox in the association between tinnitus and high levels of work- and leisure-time physical activity. Additional limitations include the self-reported nature of many variables in the analysis. Because physical activity, tinnitus, noise exposure, and depressive symptoms were all self-reported, the observed associations may be subject to same source (common method) bias. Occupational noise exposure, in particular, is susceptible to recall bias and is likely underreported, which may have limited the effectiveness of statistical adjustment for this important confounder. Nevertheless, reliance on self-reported measures is common in large population-based studies and allows associations to be uncovered that merit further investigation. In addition, the outcome reflected past-12-month prevalent self-reported tinnitus rather than incident tinnitus, precluding inferences about tinnitus onset. Some areas for further investigation based on the present study include objective measures of physical activity, specific types of occupational activity rather than general physical activity categories, and more comprehensive measures of allostatic load and stress. Exploring these areas may better characterize the influence of WORK PA on tinnitus development.

## 5. Conclusions

The present study provides population-level evidence of a domain-specific paradox in the association between physical activity and tinnitus among U.S. adults. Using NHANES 2015–2018 data, we found that high NW PA was associated with significantly reduced odds of tinnitus, while high WORK PA was associated with significantly elevated odds, a divergence confirmed by opposing linear trends across ordered categories in both domains, though the NW PA findings were borderline significant and should be interpreted cautiously pending replication. These findings align with emerging evidence from cardiovascular and inflammatory research that occupational and leisure-time physical activity are not physiologically equivalent and suggest that this distinction extends to auditory health outcomes. Collectively, these findings underscore the need to move beyond aggregate physical activity measures when examining health outcomes and highlight NW PA as a potentially accessible, non-invasive strategy worthy of further investigation in relation to tinnitus.

## Figures and Tables

**Table 1:** Weighted sample characteristics by tinnitus status (NHANES 2015–2018).

Variable	Overall ^1^ N = 96,831,795	No Tinnitus ^1^ N = 80,052,718	Tinnitus ^1^ N = 16,779,077	*p*-Value ^2^
WORK PA				0.054
Low	2706 (58.0%)	2304 (58.7%)	402 (54.5%)	
Moderate	249 (7.2%)	206 (7.5%)	43 (5.9%)	
High	1346 (34.8%)	1093 (33.8%)	253 (39.6%)	
NW PA				<0.001 **
Low	2533 (53.4%)	2068 (51.1%)	465 (64.6%)	
Moderate	609 (16.8%)	526 (17.2%)	83 (14.9%)	
High	1159 (29.8%)	1009 (31.7%)	150 (20.4%)	
Age, years	47.9 (0.48)	46.7 (0.45)	53.7 (0.86)	<0.001 **
Sex				0.142
Male	2118 (48.5%)	1740 (47.8%)	378 (52.2%)	
Female	2183 (51.5%)	1863 (52.2%)	320 (47.8%)	
Race				0.002 **
Mexican American	693 (8.2%)	560 (8.2%)	133 (8.3%)	
Other Hispanic	512 (5.9%)	439 (6.3%)	73 (4.2%)	
Non-Hispanic White	1570 (66.9%)	1252 (65.4%)	318 (74.0%)	
Non-Hispanic Black	904 (10.5%)	789 (11.2%)	115 (7.3%)	
Other Race/Multi-racial	622 (8.4%)	563 (8.95%)	59 (6.2%)	
Education				0.036 *
High school or below	1882 (33.9%)	1532 (32.7%)	350 (39.4%)	
Some college or AA degree	1322 (32.2%)	1098 (31.9%)	224 (33.3%)	
College graduate or above	1097 (33.9%)	973 (35.3%)	124 (27.3%)	
Poverty–income ratio	3.1 (0.10)	3.0 (0.09)	3.1 (0.13)	0.447
Smoking status				0.087
Never smoker	2443 (56.2%)	2120 (57.4%)	323 (50.6%)	
Former smoker	1029 (25.4%)	819 (24.8%)	210 (28.1%)	
Current smoker	829 (18.4%)	664 (17.8%)	165 (21.3%)	
BMI	29.6 (0.24)	29.4 (0.27)	30.5 (0.41)	0.030 *
Hypertension (Yes)	1578 (32.9%)	1217 (30.3%)	361 (45.4%)	<0.001 *
Diabetes (Yes)	680 (11.9%)	534 (11.0%)	146 (16.3%)	0.004 **
Depression				<0.001 **
No	3952 (92.4%)	3362 (93.6%)	590 (86.8%)	
Yes	349 (7.6%)	241 (6.4%)	108 (13.2%)	
Noise exposure (Yes)	1769 (41.0%)	1371 (38.0%)	398 (55.4%)	<0.001 **
Sedentary time, hour/day	6.56 (0.09)	6.51 (0.10)	6.79 (0.17)	0.113

^1^ Mean (SE); n (unweighted), weighted %; ^2^ Design-based *t*-test; Pearson’s χ^2^: Rao & Scott adjustment; * *p* < 0.05, ** *p* < 0.01. N = weighted population estimate.

**Table 2:** Logistic regression analyses of the association between WORK PA and tinnitus.

Variable	OR (95% CI)	*p*-Value
WORK PA		
Low	Reference	
Moderate	0.83 (0.47–1.46)	0.476
High	1.30 (1.06–1.60)	0.018 *
Sedentary time (hour/day)	1.04 (1.002–1.082)	0.038 *
Age	1.02 (1.02–1.03)	<0.001 **
PIR	1.06 (0.99–1.14)	0.097
BMI	1.01 (0.99–1.03)	0.517
Sex		
Male	Reference	
Female	1.01 (0.81–1.27)	0.908
Race		
Mexican American	Reference	
Other Hispanic	0.61 (0.42–0.89)	0.015 *
Non-Hispanic White	0.84 (0.59–1.2)	0.300
Non-Hispanic Black	0.52 (0.37–0.74)	0.001 **
Other Race/Multi-racial	0.69 (0.44–1.07)	0.090
Noise Exposure (Yes)	1.95 (1.34–2.83)	0.002 **
Smoking		
Never smoker	Reference	
Former smoker	0.85 (0.58–1.23)	0.351
Current smoker	1.05 (0.84–1.30)	0.656
Education		
High school or below	Reference	
Some college or AA degree	0.92 (0.68–1.24)	0.561
College graduate or above	0.73 (0.42–1.28)	0.246
Hypertension (Yes)	1.28 (0.93–1.76)	0.114
Diabetes (Yes)	0.97 (0.67–1.40)	0.838
Depression (Yes)	2.03 (1.40–2.94)	0.001 **

* *p* < 0.05, ** *p* < 0.01.

**Table 3:** Logistic regression analyses of the association between NW PA and tinnitus.

Variable	OR (95% CI)	p-Value
NW PA		
Low	Reference	
Moderate	0.79 (0.53–1.17)	0.211
High	0.70 (0.488–0.995)	0.0475 *
Sedentary time (hour/day)	1.00 (0.99–1.06)	0.148
Age	1.02 (1.02–1.03)	<0.001 ***
PIR	1.07 (1.00–1.16)	0.050
BMI	1.00 (0.98–1.02)	0.657
Sex		
Male	Reference	
Female	0.97 (0.78–1.22)	0.779
Race		
Mexican American	Reference	
Other Hispanic	0.62 (0.42–0.91)	0.019 *
Non-Hispanic White	0.88 (0.61–1.25)	0.433
Non-Hispanic Black	0.54 (0.39–0.75)	0.002 **
Other Race/Multi-racial	0.68 (0.43–1.06)	0.080
Noise Exposure (Yes)	2.02 (1.40–2.91)	0.001 **
Smoking		
Never smoker	Reference	
Former smoker	0.85 (0.59–1.24)	0.363
Current smoker	1.05 (0.85–1.30)	0.631
Education		
High school or below	Reference	
Some college or AA degree	0.94 (0.70–1.27)	0.677
College graduate or above	0.76 (0.44–1.32)	0.298
Hypertension (Yes)	1.25 (0.92–1.70)	0.134
Diabetes (Yes)	0.96 (0.67–1.39)	0.832
Depression (Yes)	1.98 (1.36–2.86)	0.002 **

* *p* < 0.05, ** *p* < 0.01, *** *p* < 0.001. Note: the high NW PA *p*-value is reported to four decimals given its proximity to 0.05.

**Table 4:** Linear trend analysis of physical activity (ordered categories) and tinnitus by physical activity domain.

Physical Activity Domain	β	OR per Category Increase	*p*-Value
NW PA	−0.186	0.83 (0.696–0.991)	0.0406 *
WORK PA	0.126	1.14 (1.03–1.25)	0.017 *

* *p* < 0.05. Note: the high NW PA trend *p*-value is reported to four decimals given its proximity to 0.05.

## Data Availability

Data related to NHANES are openly available in a public repository (National Health & Nutrition Examination Survey, https://www.cdc.gov/nchs/nhanes/index.html (accessed on 5 April 2026)).
